# Gender Differences in Perceived Stress and Its Relationship to Telomere Length in Costa Rican Adults

**DOI:** 10.3389/fpsyg.2022.712660

**Published:** 2022-02-25

**Authors:** Ericka Méndez-Chacón

**Affiliations:** Centro Centroamericano de Población, Escuela de Estadística, Universidad de Costa Rica, San José, Costa Rica

**Keywords:** stress, telomeres, health, caregiving, family relationships, life styles

## Abstract

**Introduction:**

Stress is associated with disease and reduced leukocyte telomere length (LTL). The objective of this research is to determine if self-perceived stress is associated with telomere length in Costa Rican adults and the gender differences in this association. Findings may help explain how some populations in apparent socioeconomic disadvantage and with limited access to specialized medical services have a remarkably high life expectancy.

**Methodology:**

Data come from the pre-retirement cohort of the Costa Rican Longevity and Healthy Aging Study (CRELES), a population based survey conducted in the households to 2,327 adults aged 53 to 66 years. The DNA to measure LTL was extracted from blood cells in laboratories of the University of Costa Rica whereas the Blackburn laboratory at the University of California performed the telomere length measurement applying the quantitative polymerase chain reaction (Q-PCR). The relationship between telomere length and perceived stress was measured using least-squares multiple regression. Perceived stress was measured by a set of questions about family, job, finances and, health reasons to be stressed. Models included the control variables: (1) age and sex of the participant, (2) whether he or she resides in the Nicoya area, a “blue zone” known for its high longevity, and (3) the aforementioned sociodemographic, health and lifestyles characteristics.

**Results:**

Stress perception and LTL are significantly different by sex. Women perceived higher stress levels than men in almost all aspects studied, except work. Women have significantly longer telomeres. Shorter telomeres are significantly associated with caregiving stress in men and with parental health concerns in women. Counter-intuitive telomere lengthenings were observed among women who feel stressed about caring for family members; and among men who feel stressed due to their family relationships as well as concerns about their own health.

**Discussion:**

Results confirm that people with self-perceived stress due to caregiving or health issues have shorter telomeres. The relationship between stress and telomere length differs between men and women. Gender relations exert a strong modifier effect on the relationship between stress and LTL: gender is related to perceived stress, telomere length, and apparently also to the way stress and LTL are related.

## Introduction

Recent studies suggest that high-stress levels are associated to reduced telomere length in children and adults (Epel et al., 2004; Ludlow et al., 2013; Mathur et al., 2016; Schaakxs et al., 2016; Rentscher et al., 2020). Evidence to support the existence of this potentially causal relationship in populations that are not usually studied is essential to have a biomarker of stress as well as to understand the mechanisms by which stress influences people’s health and longevity since studies seem to underpin that shorter telomeres indicate greater cellular aging (Shammas, 2011; Steenstrup et al., 2013; Spivak et al., 2016; Vidaček et al., 2018; Whittemore et al., 2019). Unraveling the mechanisms by which stress influences people’s health would also help to understand how certain populations with apparent socioeconomic disadvantages and limited access to specialized medical services have a high life expectancy, as it is the case of the Hispanic population in the United States, in what is known as the Hispanic paradox (Ruiz et al., 2013), or that of the exceptional longevity region of Nicoya in Costa Rica.

Prolonged periods of stress can lead to physical illness and mental disorders (McEwen and Seeman, 1999; Goldstein and McEwen, 2002). Chronic exposure to stress hormones, called catecholamines, such as adrenaline and cortisol, during some period of life development, has been found to harm the brain structures involved in cognition and physical and mental health (Lupien et al., 2009). Negative effects of stress on methylation have also been found in various genes due to conditions associated with low socioeconomic status and its implications (Cunliffe, 2016). Animal models using mice have shown that chronic catecholamine stimulation causes systemic damage to chromosomes (Hara et al., 2011). Evidence of an association between high perceived stress and shorter telomeres has also been found (Mathur et al., 2016). Shorter telomere lengths have been linked to cellular aging and the development of numerous diseases such as diabetes, cancer, heart disease, and depression (Price et al., 2013; Ridout et al., 2015).

Telomeres are the ends of chromosomes and are composed of repetitive sequences of DNA attached to proteins. Their main function is to maintain their structural stability and prevent them from fusing together (Aubert and Lansdorp, 2008). In this way, they act as plugs that protect the internal regions. They also wear out little by little with each DNA replication (Aubert and Lansdorp, 2008). Consequently, a discontinuous chain of nitrogenous bases is generated in the chromosomes that cannot be completed, therefore an asymmetric shortening occurs (Viña et al., 2005; do Vale and Escera, 2018). Some studies have associated the effects of chronic stress with reduced transcription of the catalytic component of telomerase, known as hTERT (Steptoe et al., 2007; Choi et al., 2008). However, other studies indicate that shorter telomeres are not associated with increased psychological stress *in vivo* and cannot be explained by a direct effect of cortisol (Bull et al., 2015). The mechanism by which telomere shortening has been related to stress remains unclear.

Telomere length differs by sex, with women having longer telomeres on average. It is believed that estrogen has antioxidant properties that can protect the telomeres and that testosterone lacks these properties. Differences may also respond to the body size, assuming that a larger body, such as that of men, requires more cellular divisions (Stindl, 2004; Aviv et al., 2005; Viña et al., 2005; Barrett and Richardson, 2011; Axson et al., 2018; do Vale and Escera, 2018; Ghimire et al., 2019; Lulkiewicz et al., 2020).

Susceptibility and resilience to stress vary according to some individual characteristics such as age and gender (Kim et al., 2016; Hodes and Epperson, 2019; Brivio et al., 2020). Sexual differences in how the hypothalamic-pituitary-adrenal axis responds to stress are well established, and women tend to show more intense responses (Sze and Brunton, 2019). These differences can be explained partly by gonadal sex steroids and their neuroactive metabolites, as well as by the action of orexins, which regulate the stress response and are altered in anxious and depressed patients (Grafe and Bhatnagar, 2018). Explanations are also provided based on the social and cultural context due to gender roles and stereotypes (Mayor, 2015).

In the case of older people in Costa Rica, it has been observed that telomeres are shorter in advanced ages, males, and people with diabetes. On the other hand, telomeres are longer among people in the Nicoya region, which is known by its high longevity (Rehkopf et al., 2013; Rosero-Bixby et al., 2019). It has also been found that the length of the telomeres varies depending on the month in which the sample was taken. The shorter telomeres are associated with the rainy season and DNA storage time (Rehkopf et al., 2013, 2014; Rosero-Bixby et al., 2019).

No studies were found that relate the effect of perceived stress to telomere length in Latin American populations. Costa Rica is a Central American country whose life expectancy is higher than the world average for both sexes (82.6 years for women and 77.5 years for men) (Instituto Nacional de Estadística y Censos de Costa, 2016). It has the highest life expectancy in Latin America, slightly higher than that of the United States and very similar to that of England and Western Europe (The World Bank, 2020). Costa Rica represents an atypical case since its life expectancy corresponds to the expected level of more developed economies with a per capita GDP of approximately $40,000^1^ (Rosero-Bixby and Dow, 2016).

Costa Rican nonagenarians have an exceptionally high life expectancy, half a year longer than any other country in the world in the 1990s (Rosero-Bixby et al., 2010). Costa Rica has the region of Nicoya, identified by the National Geographic as a “blue zone” of exceptional longevity (Buettner, 2008). For a 60-year-old Nicoyan male, the probability of becoming a centenarian is seven times higher than that of a Japanese male, and his life expectancy is 2.2 years longer. Nicoyan males of this age have longer telomere length than males in the rest of the country and more favorable levels of dehydroepiandrosterone sulfate (DHEA-S) (Rosero-Bixby et al., 2013). Concentrations of DHEA-S have been seen to decline with aging and this has been associated with immune system dysfunction, increased oxidative stress and cognitive decline (Klinge et al., 2018).

According to Rentscher et al. (2020), “perceived stress has been characterized in the literature as the degree to which an individual appraises their life as stressful, including feelings of being stressed, upset or angry, and cognitions that one does not have control or that demands of a situation outweigh one’s resources.” In this study, perceived stress was measured by a set of questions about family, job, finances and, health reasons to be stressed.

Understanding the differences by sex reported in the literature in both telomere length and stress management, motivated this article. Its objective is to determine if self-perceived stress is associated with leukocyte telomere length in Costa Rican adults and if this association differs by sex. Specifically, the hypotheses to be tested are:

1) People with high self-perceived stress may have shorter telomeres.
2) The relationship between stress and telomere length differs between men and women. Among men, stress resulting from labor and financial matters affects telomere length, while among women, telomere length is associated with stress derived from family, caregiving, and health issues.

If the data show that people with high levels of self-perceived stress have shorter telomeres, this would help to understand the exceptionally high longevity observed in the country and the ability to cope with stress. Likewise, if it is found that this association presents a sex differential, it could be attributed to differences in gender roles.

The study population is Costa Rican people between ages 53–66. This age range is previous to retirement, in which the culmination of maturity occurs, and it is also the threshold to be considered an older adult. At these ages, certain important physical, psychological, and socioeconomic changes appear or are accentuated, and they need to be dealt with as people age.

## Data and Methods

### Data

The data come from the longitudinal, national, and probability surveys on the health and life experiences of older Costa Ricans conducted by the Costa Rican Longevity and Healthy Aging Study (CRELES). The study is restricted to the baseline of the CRELES retirement cohort, referred to as CRELES-RC, which corresponds to people born between 1945 and 1955 and interviewed between 2010 and 2011. CRELES databases are publicly available at http://creles-download.demog.berkeley.edu/CRdata.pl.

It is worth noting that the CRELES includes two samples of adults: (1) an older cohort of individuals born before 1946 and (2) the so called retirement cohort of adults born between 1945 and 1955. This article focuses in the retirement cohort only since the older group did not collect data on perceived stress. Measurement issues and correlates of telomere length have been studied for the older CRELES cohort elsewere (Rosero-Bixby et al., 2019).

CRELES-RC participants were selected using a multi-stage cluster sample design. In the first stage, 60 health areas were selected from a total of 120. Subsequently, 222 clusters were selected from the census database, each with at least 15 people born between 1945 and 1955. The sample included all homes in those clusters with eligible individuals. In households with several persons meeting the age requirement, one was randomly chosen. The non-response rate was 43%.

The CRELES-RC study included a structured interview, anthropometric measurements, physical functioning tests, and the collection of blood samples in participants’ households. The questionnaire included questions on marital history, childhood characteristics, health and health behaviors, income, perceived socioeconomic status, housing characteristics, intergenerational transfers, and social support. Questions were also asked about perceived stress, physical activity [IPAQ scale (Booth, 2000)], cognitive status, symptoms of depression [short version of the Yesavage Geriatric Depression Scale (Yesavage et al., 1982)], and friendships.

Both the information and the blood samples were collected at the respondent’s home. Blood samples were taken by venipuncture by certified phlebotomists and, after initial processing in the field, aliquoted and stored in laboratories of the University of Costa Rica stored in 2 ml vials at –40°C (Dow et al., 2013).

### Outcome: Leukocytes Telomere Length

Laboratories of the University of Costa Rica extracted DNA from frozen blood samples following the Hermann and Frischauf methodology (Herrmann and Frischauf, 1987). The DNA samples were sent to the Blackburn laboratory at the University of California, San Francisco for telomere length measurements, in assays conducted in 2014. This laboratory used the quantitative polymerase chain reaction (Q-PCR) to determine the telomere ratio to a single copy gene (T/S ratio), in this case, human beta-globulin. Each DNA sample was assayed three times and T and S values were averaged to obtain the T/S ratio. The average inter-assay coefficient of variability was 0.037 (0.033 s.d.) (Rosero-Bixby et al., 2019).

Since analyses of the pre-1945 CRELES cohort detected significant variations in LTL by storage times and assay lot as well as seasonal variations by month of blood collection (Rosero-Bixby et al., 2019), for the current analysis, the months that the blood and DNA were stored, as well as the month in which the blood sample was obtained, were included as control variables in the multiple regression models.

The public CRELES data files include observed and adjusted LTL values. The adjusted values control the confounding effect of variable storage times and measurement lot are estimates of what would have been obtained if DNA samples were extracted from blood cells stored less than 12 months and the LTL assays were conducted immediately after DNA extraction (zero storage time of DNA) using the procedures of the 2014 assay (Rosero-Bixby et al., 2021). In order to make the LTL indicator comparable across all waves, for CRELES-RC, the current analysis uses the adjusted LTL values (Adj_T/S = Obs_T/S −0.305).

### Factor of Interest: Perceived Stress

Perceived stress was measured in the same way as in Taiwan Longitudinal Study on Aging (TLSA) (Vasunilashorn et al., 2013, 2015; Vasunilashorn and Cohen, 2014; Tsai et al., 2015) by the following questions about reasons to be stressed out:

• Work*:* Problems at your **job**; do they make you feel stressed or anxious?
• Family: Do **your family relationships** make you feel stressed or anxious?
• Own Health: Does your **health** make you feel stressed or anxious?
• Finances: Does your **financial situation** make you feel stressed or anxious?
• Relatives’ Health: Does the **health of your parents** or other family members make you feel stressed or anxious?

When people answered yes to any of these questions, they were asked how long they had been feeling stressed. The response options were for more than a year or less than a year. In the case of people who were not doing paid work outside the home (1,269 people), it was considered that they did not have stress related to their work. Most of these cases were women. Also, 13 people stated that they had no family. They were also considered to have no stress due to family relationships or the health of their parents.

Additionally, they were asked about the perceived stress of **helping a family member** with basic activities such as dressing, eating, or bathing him/her, due to a health condition. If for the purposes of this study, people were not helping any family member, they were considered as being free of stress in this regard. This is called “caregiving stress.”

### Covariates

**Sex:** Male and female.

**Age in years:** Age was verified against the official identification document.

**Nicoya:** Indicates if people are residents of Nicoya, a “blue zone” known for its high longevity.

**Marital Status:** Dichotomized as have a partner (by marriage or free union) and not have a partner.

**Education:** Dichotomized as primary education or lower and secondary education or higher.

**Self-reported health:** It was recorded if people themselves reported having been diagnosed as having any of the following chronic diseases: high blood pressure, cholesterol, diabetes, cancer, lung disease, heart attack, other heart diseases, stroke, arthritis, osteoporosis, cataracts, and depression. It was also recorded how the respondent perceived his or her health condition by using the standard scale of (1) excellent, (2) very good, (3) good, (4) fair, and (5) poor.

**Social Support Index:** Adults were given a battery of questions regarding the support they receive in different situations. Each item is measured on a scale of 1 to 4, where 1 corresponds to never happening and 4 happens very frequently. Items are:

• You are invited you to distract yourself and go out with other people.
• You receive love and affection.
• You can talk to someone about your personal and family problems.
• You can talk to someone about your problems at work or home.
• You can talk to someone about your financial problems.
• You have people who care about what happens to you.
• You receive useful advice when an important event in your life happens to you.
• You get help when you are sick in bed.

An indicator, ranging 8–32, was computed by adding the answers obtained in these items.

#### Health Risk Behavior Indicators

• Smoking status: smokes or has ever smoked (includes active and ex-smokers) and never smoked.
• Alcohol consumption: ever consumed liquor (includes daily drinker, occasional drinker, and ex-drinker) and never drunk.
• Physical activity: physical activity was measured using the IPAQ scale and people were grouped according to their physical activity in two categories as mild, and moderate or high. Mild category includes people that report no regular physical activity. Moderate or high correspond to people who walk at least 30 minutes per day or perform more vigorous excecise.

**Anthropometric measurements:** Body mass index and knee height were used as an indication of the physical composition and state of child development, respectively. The body measurements were taken by trained fieldworkers using “Life Source, M&D medical scales, model UC-321p”, Seca stadiometers. Knee height was measured on the right leg; the leg was placed at a 90-degree angle with help of a goniometer and then the length was measured with Shorr USES Knee-Height Calipers.

### Ethical Aspects

The Scientific Ethics Committee of the University of Costa Rica (references: VI-2878-2009, VI-5308-2009, and VI-1313-2012), approved the CRELES research project. The study of the retirement cohort was also approved by the Committee for Protection of Human Subjects (CPHS II) at the University of California, Berkeley. Written informed consent was signed by each participant during the first round of interviews. All data have been anonymized.

### Statistical Analyses

Several two tailed *t*-tests were conducted for each stress domain to examine differences in LTL for participants who reported stress vs. no stress (Supplementary Table 2). Chi square test were also applied to compare differences in category variables between males and females and 95% confidence intervals for mumeric variables. All these tests were estimated using the svy command at Stata software (Stata/MP version14.1). A factor analysis (with a tetrachoric correlations matrix) was performed to determine the structure of the relationships between the variables that measured stress and the adequacy of combining them into a single factor. Cronbach’s Alpha was calculated as an empirical measure of the reliability of a scale summarizing the stress variables.

Then, a separate multiple regression model was conducted, one for females and another for males. The dependent variable was LTL, measured by the T/S ratio. Models included the control variables: (1) age of the participant, (2) whether he or she resides in the Nicoya area —since it is already known that they are related to telomere length in slightly longer-living Costa Rican populations (Rehkopf et al., 2013), (3) the aforementioned sociodemographic, health and lifestyles characteristics and, (4) all variables related to the perception of stress, which are this article’s factor of interest. Dummy variables were created to compare “stress for more than a year” and “stress less than a year” to “no stress.” The reason to fit separate models by gender (instead of a single model with gender interactions) is that LTL and stress perception differ substantially by gender according to the literature (Aviv et al., 2005; Axson et al., 2018; Hodes and Epperson, 2019; Brivio et al., 2020).

CRELES study has a complex sample design, to analice this data, we must consider the weighting, clustering, and stratification of the survey design to get the standard errors right. All calculations were adjusted considering the complex sampling design and the weighing factors, as implemented in the Stata software. Ignoring the design elements that are included can lead to inaccurate point estimates and/or inaccurate standard errors.

## Results

### Descriptive Analysis

Sample size of CRELES-RC is 2,798 participants; however, this study used only the information of participants with the datum of telomere length for an analytical sample size of 2,327.

Table 1 summarizes the general characteristics of the sample studied. Of them, 931 or 40% are men. The average age is 59 years, range: 53–66 years. Six percent live in Nicoya. Sixty percent of women and 84% of men reported living with a partner. Only 40% of participants reported some level secondary education attainment.

**TABLE 1:** General characteristics (averages or proportions) by sex.

Variables (*n*)	Units	Males (*n* = 931)			Females (*n* = 1,396)		
			
		Mean	SE	*n* *	Mean	SE	*n* *
Demographics and socioeconomics							
Nicoya region (*n* = 2327)	Binary 0-1	0.06	0.02	69	0.06	0.02	97
Age (*n* = 2327)	Years	59.62	0.15	931	59.39	0.12	1396
Marital status (*n* = 2327)	Binary 0-1						
Married or free union		0.85	0.01	741	0.60	0.02	765
Other		0.16	0.01	190	0.40	0.02	631
Level of education (*n* = 2327)	Binary 0-1						
Elementary		0.57	0.03	612	0.58	0.03	963
Secondary or higher		0.44	0.03	319	0.42	0.03	433
Self-reported health (*n* = 2327)	Binary 0-1						
Hypertension		0.40	0.02	367	0.53	0.02	754
Cholesterol		0.36	0.02	333	0.52	0.02	755
Diabetes		0.16	0.02	155	0.19	0.01	283
Cancer		0.04	0.01	32	0.06	0.01	84
Pulmonary disease		0.08	0.01	87	0.17	0.02	237
Heart attack		0.03	0.01	29	0.03	0.01	38
Other heart diseases		0.07	0.01	68	0.11	0.01	154
Stroke		0.01	0.01	10	0.01	0.00	13
Arthritis		0.04	0.01	48	0.14	0.01	198
Osteoporosis		0.01	0.00	12	0.11	0.01	145
Cataracts		0.18	0.02	184	0.16	0.01	234
Mental disorders		0.13	0.01	130	0.27	0.02	384
Poor self-reported health		0.39	0.03	416	0.48	0.02	704
Lifestyles (*n* = 2327)							
Social support index	Scale 8-32	24.89	0.23	931	25.86	0.23	1396
Smoker condition	Binary 0-1						
Sometimes		0.64	0.02	594	0.21	0.02	276
Never		0.36	0.02	337	0.79	0.02	1120
Alcohol consumption	Binary 0-1						
Sometimes		0.95	0.01	886	0.52	0.02	703
Never		0.05	0.01	45	0.48	0.02	693
Physical activity (*n* = 2306)^1^	Binary 0-1						
Low		0.34	0.02	291	0.40	0.02	569
Moderate or high		0.66	0.02	632	0.60	0.02	814
Biomarkers							
Body mass index (*n* = 2314)	Kg/m^2^	26.97	0.19	1390	29.34	0.20	924
Knee height (*n* = 2306)	Cm	51.37	0.13	919	47.37	0.09	1387

*Estimates include corrections for complex sampling design and the weighing factors.*

*Unweighted.

*^1^Males (n = 923), Females (n = 1383).*

The prevalence of chronic diseases is gender-differentiated (Table 1) and is higher in women than in men. The most prevalent diseases were metabolic diseases such as hypertension, cholesterol, and diabetes. Also, a greater proportion of women perceived worse health conditions (48% of women compared to 39% of men).

There are also differences by gender in relation to lifestyle. For example, 79% of women say they have never smoked, compared to 36% of men. Concerning alcohol consumption, only 5% of men mentioned they had never consumed alcohol, while women’s percentage is 48%. More than 60% out of the total reports some degree of physical activity. An important aspect is that both men and women have a strong social support network (an average of 25 points out of a maximum of 32). Finally, the results of the biomarker show that the average body mass index is within the overweight range, especially in women.

### Perceived Stress

**FIGURE 1:**
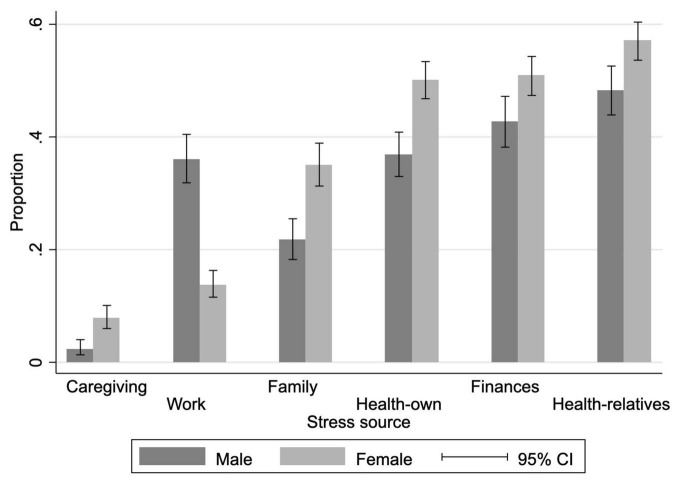
Proportion of people who feel stressed, by source of stress and sex. Estimates include corrections for complex sampling design and the weighing factors. All pairs of comparisons by the source of stress are significant at 1%.

Most participants reported feeling stress related to concerns about the health condition of their parents and relatives, their financial situation, and their own health. The perception of stress was significantly different by gender. Women perceived higher levels of stress than men in almost all aspects studied, except for work, possibly because many of them do not work outside their home (Figure 1). For example, 57% of women perceived having suffered from stress due to their parents’ or other family members’ health compared to 48% of men. This pattern of a higher percentage of women perceiving stress is independent of whether people report feeling stressed less than a year ago or for more than a year (Supplementary Table 1).

These six sources of perceived stress are grouped into a factor that explains 70% of the variability. So, these items comply with the one-dimensionality of a measurement instrument assumption. Cronbach’s alpha value as a measure of internal consistency is 0.55. It increases to 0.57 if the item of stress due to caring for sick people is eliminated. After observing these indicators, the stress domains were analyzed separately.

### Telomere Length According to Perceived Stress

The average length of the telomeres in leukocytes (LTL), measured by the T/S ratio, was 0.95 (SE: 0.008) in the entire population. The LTL differs significantly by sex. Women’s telomeres were significantly longer (0.97; SE: 0.01) than men’s (0.92; SE: 0.01).

**FIGURE 2:**
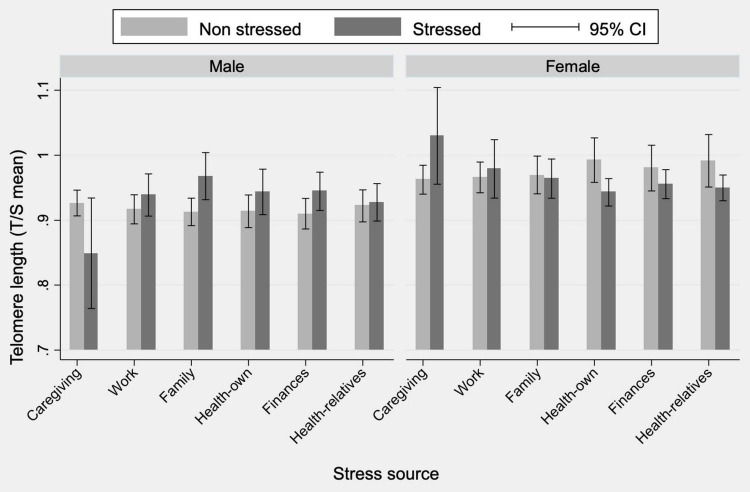
Mean telomere length by source of stress and sex. Estimates include corrections for complex sampling design and the weighing factors. Differences in mean telomere length due to family stress in men are significant at 1%. Differences in mean telomere length due to own health stress in women are significant at 1%.

In a preliminary analysis without distinction by sex, no differences were found in telomere length between those who perceived stress and those who did not (Supplementary Table 2). However, significant differences emerged when analyzing men and women separately and for certain types of self-perceived stress, as shown in Figure 2.

Shorter telomeres are associated with stress only in stress derived from caring for sick relatives in the case of men and that derived from concerns about one’s own health, financial issues, and parental health in the case of women. In contrast, a counter-intuitive telomere lengthening is observed among women who feel stressed about caring for family members. These associations are apparently little or not significant since the confidence intervals between stress and non-stress overlap.

### Multivariate Analysis of the Leukocyte Telomere Length and Stress Relationship

The results of univariate analyses described so far might be confounded by the peculiarities of the people subject to stress. For example, if female caregivers are younger, they will have longer LTLs, and this might hide an eventual telomere shortening effect associated with this type of stress. Additionally, it is necessary to make a more precise and efficient estimate of the statistical significance of the differences in LTL beyond the simple observation of overlapping confidence intervals. The following multivariate approach using linear regression models addresses these issues. The models also include as a potentially explanatory factor, akin to a dose-response effect, the amount of exposure time since the reported stress event as another explanatory dimension. Table 2 summarizes the results obtained with these regression models.

**TABLE 2:** Regression coefficients that describe the relationship between telomere length and stress perception by sex.

Variables	Males (*n* = 911)				Females (*n* = 1,375)			
		
	Coefficient	95% conf. interval			Coefficient	95% conf. interval		
Take care of sick relatives	−0.11	−0.19	−0.03	**	0.09	0.03	0.15	**
Own health (reference without stress)								
Less than a year	0.06	0.00	0.12	*	−0.04	−0.08	0.00	*
More than one year	0.02	−0.03	0.08		−0.02	−0.06	0.01	
Financial situation (reference without stress)								
Less than a year	0.03	−0.03	0.09		0.01	−0.03	0.05	
More than one year	0.00	−0.04	0.05		−0.01	−0.04	0.03	
Work problems (reference without stress)								
Less than a year	0.00	−0.06	0.06		0.07	0.00	0.13	
More than one year	0.00	−0.04	0.05		−0.03	−0.08	0.02	
Family relationships (reference without stress)								
Less than a year	0.02	−0.05	0.09		0.05	−0.02	0.12	
More than one year	0.06	0.01	0.11	**	0.00	−0.03	0.03	
Relatives health (reference without stress)								
Less than a year	0.05	−0.01	0.11		−0.02	−0.08	0.03	
More than one year	−0.02	−0.06	0.03		−0.05	−0.09	−0.02	**

*Estimations correspond to weighted samples using complex sample design.*

*Variables from Table 1 as well as months that the blood and DNA were stored and month in which the blood sample was obtained, were also included in the models, but they are not shown.*

*Significant at 0.05 level.

**Significant at 0.01 level.

The analysis with multiple regression confirms the main univariate results presented above, i.e., relationships between certain types of stress and telomere length, and that these relationships are differential by sex. In the case of men, those who perceive stress from caring for sick relatives present a significant decrease in telomere length. Paradoxically, those who felt stressed for more than a year due to family relationships have longer telomeres on average than other men who do not feel stress related to family issues.

In the case of women, those who perceive recent stress (less than a year) due to their own health condition, or for feeling prolonged stress (more than a year) related to the health issues of their parents or relatives, present a significant decrease of 0.04 in telomere length. Unexpectedly, contrary to men, an increase in telomere length was observed among women who perceived stress from care for sick relatives with respect to those who did not report stress (*p*-value <0.05).

## Discussion

Hypothesis 1 that people with self-perceived stress have shorter telomeres, was corroborated with data only for men who feel stressed about caring for sick relatives and for women who feel stressed due to their own health issues and the health condition of parents or relatives. No significant association was found between telomere length and financial stress. And the association was contrary to the hypothesis for men who feel stress from family relationships or their own health and women who feel stress from caring for family members.

Hypothesis 2, which states that the relationship between stress and telomere length differs between men and women, was confirmed by the data, both by the differences noted in the previous paragraph and by the fact that significant relationships between perceived stress and telomere length emerge only by performing the analysis disaggregated by sex. In other words, gender relations have a strong modifier effect on the relationship between stress and LTL, as gender is related to perceived stress, telomere length, and apparently also to how stress and LTL are related.

Likewise, the data confirmed that women have significantly longer telomeres than men in this population. This result is similar to that found among elderly Costa Ricans (Rosero-Bixby et al., 2019) and is consistent with studies performed in other populations around the world, such as those conducted in the United States (Gardner et al., 2014). The reasons why telomeres are longer in women are still under investigation. Some authors suggest that telomere length is linked to the X chromosome (Nawrot et al., 2004), or that estrogen may exert an antioxidant effect that protects against the DNA damage induced by reactive oxygen species (ROS) in addition to stimulating telomerase (Aviv, 2002).

Data confirmed that women in Costa Rica report more stress than men as reported from women in U.S. populations (Cohen and Janicki-Deverts, 2012). This phenomenon is widely known (Lazarus and Folkman, 1984) and some authors explain it as related to the context and the social construction of ideas, beliefs, and cultural, economic and political features that cultures generate in terms of gender roles and stereotypes (Mayor, 2015). Costa Rica is thus not an exception.

The association between stress and short telomeres from caring for sick relatives was found in men, but not in women. Other studies also report that caregivers have shorter telomeres (Litzelman et al., 2014). Yet another research suggests that telomere length is affected more by the duration of the stressor than simply the state of being or not being a caregiver (Epel et al., 2004). The differences in the relationships between telomere length and caregiving stress observed between men and women may be attributed to the same gender and role differences involving different coping strategies or stress management, integration of stressful life events and resilience, as well as the intensity of perceived stress, which was not measured in this study.

Telomeres in women were found to be shorter among those who perceived recent stress derived from their health condition (within a year or less). The associations remained after adjusting for several important variables such as lifestyle and disease conditions, among others. This suggests that the links are independent, and perhaps the perception of stress due to one’s health reflects the important impact of other elements such as serious or complex health conditions, which were not measured. Similar results have been found in the U.S. black population, whose negative perception of overall self-reported health and days of discomfort due to recent poor physical health were associated with shorter telomeres (Khan et al., 2017). If, according to Cohen et al. (2003), subjective well-being and positive emotions are associated with improved immune function in the case of common infections such as colds (Cohen et al., 2003), the question could be raised whether a reverse effect occurs with other diseases since a significant percentage of these women self-report several chronic diseases and have a poor perception of their health, thus affecting LTL. The opposite relationship is observed in men.

Telomeres are also shorter in women who have felt stressed or worried about the health conditions of their parents or family members, but for more than a year. This fact may respond more to the duration of the stress than simply to the poor health factor of the family member since physical and psychological strain may lead to exhaustion —a condition that causes the person to feel fatigued, apathetic. It may also be related to the inability to help such a close family member recover his or her health. Among caregivers (Litzelman et al., 2014),findings reveal an association between the hours of care provided per week, the younger the age of the recipient of care, and the greater tension the caregiver has, with a shorter telomere length.

On the other hand, feeling stress due to short-term work problems in the case of women is marginally significant (*p*-value = 0.05) related to an increase in telomere length. This finding is contrary to expectations since literature supports the existence of adverse health outcomes associated with shorter telomeres connected to working conditions such as the relationship between cardiovascular disease and occupational stress (Landsbergis et al., 2003). In contrast, Parks et al. (2011) showed that women aged 35–74 with full-time jobs have shorter telomeres compared to those who work part-time or not at all. In this case, since the stress is experienced in a short period of time, it could be that they are active women and that the stress they are perceiving is due to a fortuitous situation with a little time exposure to generate an effect at the telomere level.

An important aspect to highlight is that a non-stress response was attributed to 62% women because they stated having no job. By restricting the regression model only to women in the workforce (*n* = 519), the estimated effect of stress on LTL in working women that perceive stress for less than a year is a significant 0.079 [CI: 0.017–0.143] compared to to women that non-perceive stress.

According to the literature reviewed, people with a history of poor interpersonal relationships are more likely to demonstrate negative health outcomes (Murdock et al., 2018). Concerning children, telomeres are sensitive to adversity within the general family domain (Drury et al., 2012, 2014; Non et al., 2016) and low social support is associated with shorter LTL in adults (Carroll et al., 2013; Rentscher et al., 2020). Results in this last population were unexpected, as they show that men who perceive prolonged stress due to family relationships have longer telomeres.

The mechanism by which stress may be related to the gender dimension of telomeres is not clear and requires further research. The effect of stress on the immune system and the relationship that may exist between cortisol and telomerase may explain the presence of shorter telomeres. However, this mechanism could be partly influenced by psychological and personality aspects, stress evaluation skills, and the ability to judge threatening or challenging factors that, in sum, provide an individual differential coping ability, with the possibility of triggering negative response processes.

A limitation of this research is that the associations observed are of a cross-sectional type, with considerable likelihood that some of them are the result of personal characteristics that were not measured or controlled, as well as of reverse causality. For example, healthier people with longer telomeres might likely be more involved in family problems or in caregiving and thus feel more stress, which is why we would not be observing the expected association of shorter telomeres with stress. One way to avoid this type of problem would be through longitudinal data. The cross-sectional design of the study does not allow us to reach causal conclusions between the stress-telomere relationship.

Another limitation is that stress is a complex construct and was addressed by this study through simple questions. The CRELES study data were collected in a multipurpose survey with international comparative purposes over many topics within the family of Health and Retirement Surveys methodology such as the Well-Being and Aging in Latin America and the Caribbean Study (SABE) (Albala et al., 2005; Pelaez et al., 2006; Lebrão et al., 2019), Mexican Health And Aging Study (MHAS) (Wong et al., 2017) and Puerto Rican Elderly: Health Conditions Project (PREHCO) (Palloni et al., 2013).

These studies are designed to be comparable between them, and they do not use scales to measure stress such as Stress Appraisal Measure (SAM) (Peacock and Wong, 1990), the Impact of Event Scale (IES) (Sundin and Horowitz, 2003), and the Perceived Stress Scale (PSS) (Cohen et al., 1983). The battery of questions related to stress follows the Taiwan Longitudinal Study on Aging (TLSA) (Ministry of Health and Welfare, 2015). A similar Cronbach’s Alpha to those observed in Taiwan (Cronbach’s alpha for the three waves were 0.68 in 1999, 0.65 in 2003, and 0.64 in 2007) was found in Costa Rica (Vasunilashorn et al., 2013, 2015). In addition, the known perceived stress scales have not been tested in the Costa Rican population or in this specific age group. Thus, these questions are not being treated as a scale in this study.

Multi-testing is to some extend an issue. In the regression models analyzed, a total of 48 significance tests were performed associated to variables’s betas. It is likely that at a significance level of *P* 0.05 a couple of significant results occurred by chance alone. New results after using Benjamini-Hochberg corrections for multi-testing (Supplementary Table 3) do not differ meaningfully.

A strength of this study is to be based in a nationally representative sample of a Latin American country with exceptional longevity conditions. The topic addressed has not been studied in Latin America. Moreover, the size of the sample allows detecting small effects and provides a richness of relevant covariates that could have masked relationships between stress and telomeres.

## Data Availability Statement

The datasets presented in this study can be found in online repositories. The names of the repository/repositories and accession number(s) can be found below: http://creles-download.demog.berkeley.edu/CRdata.pl.

## Ethics Statement

The studies involving human participants were reviewed and approved by The Scientific Ethics Committee of the University of Costa Rica (references: VI-2878-2009, VI-5308-2009, and VI-1313-2012), approved the CRELES research project. The study of the retirement cohort was also approved by the Committee for Protection of Human Subjects (CPHS II) at the University of California, Berkeley. Written informed consent was signed by each participant during the first round of interviews. All data have been anonymized. The patients/participants provided their written informed consent to participate in this study.

## Author Contributions

EM-C contributed to conception and design of the study, organized the database, performed the statistical analysis, and wrote the manuscript.

## Conflict of Interest

The author declares that the research was conducted in the absence of any commercial or financial relationships that could be construed as a potential conflict of interest.

## Publisher’s Note

All claims expressed in this article are solely those of the authors and do not necessarily represent those of their affiliated organizations, or those of the publisher, the editors and the reviewers. Any product that may be evaluated in this article, or claim that may be made by its manufacturer, is not guaranteed or endorsed by the publisher.

## Footnotes

1 The Gross Domestic Product per capita for Costa Rica, current prices (PPP) was approximately $ 19,000, in 2017. http://hdr.undp.org/en/indicators/194906
