# Supplementary material for: Gender Differences in Perceived Stress and Its Relationship to Telomere Length in Costa Rican Adults
Source: Front Psychol. 2022 Feb 25;13:712660. doi: 10.3389/fpsyg.2022.712660 (PMC8915848; doi:10.3389/fpsyg.2022.712660)
Supplement: Supplementary file 1 [file Table_1.docx]

1. **ANNEX**

**Table A.1. Telomere length and perceived stress by sex.**

| **Variables** | **Units** | **Males (n=931)** | |  | **Females (n=1396)** | |  | **p-value** |
| --- | --- | --- | --- | --- | --- | --- | --- | --- |
|  |  | **Proportion** | **SE** | **n**** | **Proportion** | **SE** | **n**** |  |
| Telomere length (mean)^a/^ | T/S | 0.93* | 0.01 | 931 | 0.97* | 0.01 | 1396 | 0.00 |
| Stress resource ^b/^ |  |  |  |  |  |  |  |  |
| Caregiving | Binary 0-1 | 0.02 | 0.01 | 18 | 0.08 | 0.01 | 100 | 0.00 |
| Own health | Category |  |  |  |  |  |  | 0.00 |
| Without stress |  | 0.63 | 0.02 | 561 | 0.50 | 0.02 | 695 |  |
| Less than a year |  | 0.10 | 0.01 | 100 | 0.17 | 0.01 | 221 |  |
| More than one year |  | 0.27 | 0.02 | 270 | 0.33 | 0.02 | 480 |  |
| Financial situation | Category |  |  |  |  |  |  | 0.03 |
| Without stress |  | 0.57 | 0.02 | 509 | 0.49 | 0.02 | 664 |  |
| Less than a year |  | 0.11 | 0.02 | 94 | 0.13 | 0.01 | 185 |  |
| More than one year |  | 0.32 | 0.02 | 328 | 0.38 | 0.02 | 547 |  |
| Work problems | Category |  |  |  |  |  |  | 0.00 |
| Without stress |  | 0.64 | 0.02 | 590 | 0.86 | 0.01 | 1203 |  |
| Less than a year |  | 0.10 | 0.01 | 96 | 0.05 | 0.01 | 72 |  |
| More than one year |  | 0.26 | 0.02 | 245 | 0.09 | 0.01 | 121 |  |
| Family relationships | Category |  |  |  |  |  |  | 0.00 |
| Without stress |  | 0.78 | 0.02 | 718 | 0.65 | 0.02 | 915 |  |
| Less than a year |  | 0.05 | 0.01 | 39 | 0.08 | 0.01 | 100 |  |
| More than one year |  | 0.17 | 0.02 | 174 | 0.28 | 0.02 | 381 |  |
| Health of parents or relatives | Category |  |  |  |  |  |  | 0.00 |
| Without stress |  | 0.52 | 0.02 | 478 | 0.43 | 0.02 | 619 |  |
| Less than a year |  | 0.14 | 0.02 | 122 | 0.14 | 0.01 | 201 |  |
| More than one year |  | 0.34 | 0.02 | 331 | 0.43 | 0.02 | 576 |  |

**Estimates consider the complex sampling design and the weighing factors**.

***Mean**

****Unweighted**

^a/^ **P value for two-tailed t-test**

^b/^ **P value for one-tailed chi-test**
