# Supplementary material for: Gender Differences in Perceived Stress and Its Relationship to Telomere Length in Costa Rican Adults
Source: Front Psychol. 2022 Feb 25;13:712660. doi: 10.3389/fpsyg.2022.712660 (PMC8915848; doi:10.3389/fpsyg.2022.712660)
Supplement: Supplementary file 2 [file Table_2.docx]

1. **ANNEX**

**TABLE A.2. Mean telomere size according to stress condition.**

| Source | Stressed | | | | | | P-value |
| --- | --- | --- | --- | --- | --- | --- | --- |
|  | Yes | | | No | | |  |
|  | Mean | SE | n* | Mean | SE | n* |  |
| Caregiving | 0.99 | 0.03 | 118 | 0.94 | 0.01 | 2209 | 0.166 |
| Own health | 0.94 | 0.01 | 1071 | 0.95 | 0.01 | 1256 | 0.712 |
| Financial situation | 0.95 | 0.01 | 1154 | 0.94 | 0.01 | 1173 | 0.561 |
| Work problems | 0.95 | 0.01 | 534 | 0.95 | 0.01 | 1793 | 0.727 |
| Family relationships | 0.97 | 0.01 | 694 | 0.94 | 0.01 | 1633 | 0.061 |
| Health of parents or relatives | 0.94 | 0.01 | 1230 | 0.95 | 0.01 | 1097 | 0.300 |

**Estimates consider the complex sampling design and the weighing factors**.

***Unweighted**

**P value for two-tailed t-test**
