# Supplementary material for: Gender Differences in Perceived Stress and Its Relationship to Telomere Length in Costa Rican Adults
Source: Front Psychol. 2022 Feb 25;13:712660. doi: 10.3389/fpsyg.2022.712660 (PMC8915848; doi:10.3389/fpsyg.2022.712660)
Supplement: Supplementary file 3 [file Table_3.docx]

1. **ANNEX**

**TABLE A.3. Benjamini-Hochberg corrections.**

| **Variables** | **Males (n=911)** | | |  | **Females (n=1375)** | | |  |
| --- | --- | --- | --- | --- | --- | --- | --- | --- |
|  | **P value** | **Rank** | **(i/m)Q** | **Is p<(i/m)Q?** | **P value** | **Rank** | **(i/m)Q** | **Is p<(i/m)Q?** |
| Take care of sick relatives | 0.009 | 2 | 0.006 | No | 0.005 | 4 | 0.013 | Yes |
| Own health (reference without stress) |  |  |  |  |  |  |  |  |
| Less than a year | 0.045 | 10 | 0.031 | No | 0.043 | 6 | 0.019 | No |
| More than one year | 0.424 | 27 | 0.084 | No | 0.189 | 18 | 0.056 | No |
| Financial situation (reference without stress) |  |  |  |  |  |  |  |  |
| Less than a year | 0.335 | 24 | 0.075 | No | 0.682 | 42 | 0.131 | No |
| More than one year | 0.832 | 43 | 0.134 | No | 0.642 | 40 | 0.125 | No |
| Work problems (reference without stress) |  |  |  |  |  |  |  |  |
| Less than a year | 0.958 | 47 | 0.147 | No | 0.055 | 8 | 0.025 | No |
| More than one year | 0.828 | 41 | 0.128 | No | 0.19 | 19 | 0.059 | No |
| Family relationships (reference without stress) |  |  |  |  |  |  |  |  |
| Less than a year | 0.513 | 33 | 0.103 | No | 0.145 | 14 | 0.044 | No |
| More than one year | 0.012 | 5 | 0.016 | Yes | 0.997 | 48 | 0.150 | No |
| Relatives health (reference without stress) |  |  |  |  |  |  |  |  |
| Less than a year | 0.121 | 16 | 0.050 | No | 0.347 | 26 | 0.081 | No |
| More than one year | 0.453 | 31 | 0.097 | No | 0.002 | 3 | 0.009 | Yes |
| Q=0.15; m=48 |  |  |  |  |  |  |  |  |
